# *BRCA1/2* variant landscape and clinical correlates in high-risk breast cancer patients from Eastern China

**DOI:** 10.3389/fonc.2026.1792634

**Published:** 2026-06-29

**Authors:** Wei Gu, Najeeb Ullah Khan, Huijun Lei, Jinzhen Fu, Chengyin Xu, Xukai Chen, Xiao-jia Wang, Tianhui Chen

**Affiliations:** 1 Postgraduate Training Base Alliance of Wenzhou Medical University (Zhejiang Cancer Hospital), Wenzhou, China; 2 Department of Cancer Prevention, Zhejiang Cancer Hospital, Hangzhou, China; 3 Hangzhou Institute of Medicine (HIM), Chinese Academy of Sciences, Hangzhou, China; 4 Institute of Biotechnology and Genetic Engineering, The University of Agriculture Peshawar, Peshawar, Pakistan; 5 Department of Breast Medical Oncology, Zhejiang Cancer Hospital, Hangzhou, China

**Keywords:** *BRCA1/2* variant spectrum, China, hereditary breast cancer, high-risk, precision prevention

## Abstract

**Introduction:**

Global advances in genetic testing are reshaping breast cancer management. Although large unselected Chinese cohorts have been reported, the *BRCA1* and *BRCA2* (*BRCA1/2*) variant landscape specifically in high-risk, clinically referred populations remains poorly characterized. We aimed to delineate the landscape of *BRCA1/2* variants in high-risk Chinese breast cancer patients, consistent with the clinical referral and testing pathway, and to provide evidence to inform risk-adapted clinical practice for high-risk patients with breast cancer in eastern China.

**Methods:**

We analyzed 1,262 high-risk Chinese breast cancer patients using targeted next-generation sequencing to detect *BRCA1/2* germline variants. Variant clinical significance was interpreted primarily based on ClinVar annotations within the ACMG–AMP 2015 framework, with population frequency and in silico prediction results used as supporting evidence. Statistical analyses were performed in R (version 4.5.0).

**Results:**

In total, 124 cases were identified as carriers of 81 *BRCA1/2* P/LP (pathogenic or likely pathogenic) variants. P/LP carriers were more frequent in patients aged ≤45 years in our cohort. A total of 9.8% of patients carried *BRCA1/2* P/LP variants, with 5.5% and 4.3% carrying *BRCA1* and *BRCA2* variants (70 vs. 54 cases), respectively. Among the 336 patients with triple-negative breast cancer (TNBC), 60 cases carried *BRCA1/2* P/LP variants (51 *BRCA1* vs. 9 *BRCA2*). A total of 21 variants were observed in more than one participant, including *BRCA1* c.5470_5477del, a founder mutation in Chinese populations.

**Conclusion:**

Taken together, this study provides the most comprehensive characterization to date of the *BRCA1/2* variant landscape in a high-risk Chinese breast cancer cohort from mainland China, offering population-specific reference data to refine genetic counseling, screening prioritization, and risk management, with potential implications for targeted prevention and intervention in Chinese patients seen in clinical practice.

## 1 Introduction

Breast cancer is a major global health challenge, and its incidence and mortality continue to rise in China (1), indicating an urgent need to improve the effectiveness of risk assessment and prevention strategies.

Approximately 5%–6% of patients with breast cancer carry pathogenic variants in population-based studies, and *BRCA1* and *BRCA2* (*BRCA1/2*) are among the genes most frequently involved (2). *BRCA1/2* pathogenic variant carriers face markedly elevated breast cancer risk, reaching approximately 70% by the age of 80 (3), along with increased risks of ovarian and other cancers.

Although several large Chinese and East Asian cohorts have reported *BRCA1/2* variants, important gaps remain in the systematic annotation, detailed analyses of high-risk subgroups, and integration with international databases. In clinical practice in China, *BRCA1/2* testing is largely guided by professional recommendations (e.g., early onset disease or a relevant family history among patients) (4) but is not covered by medical insurance, and population-wide testing has not yet been routinely implemented in China (5). Evidence is still needed to identify populations that should be tested and to support the development of strategies for targeted secondary prevention.

We conducted the largest study of a high-risk patient cohort reported to date in mainland China, including 1,262 high-risk breast cancer patients. We aimed to delineate the landscape of *BRCA1/2* variants in high-risk Chinese patients with breast cancer and to provide specific evidence for high-risk patients with breast cancer in eastern China. In addition to annotations from the NCBI clinical variant database (ClinVar), supported by in silico predictions, we incorporated the Genome Aggregation Database, East Asian subset (gnomAD EAS) to complement variant interpretation. This approach was motivated by prior evidence of ancestry-related discrepancies (6) and the lack of publicly available large-scale genomic data in China. Our findings offer population-specific reference data that align with the high-risk clinical referral pathway, supporting more precise genetic counseling, prioritization of genetic testing and screening, and risk management for precision prevention interventions in Chinese high-risk breast cancer patients.

## 2 Materials and methods

### 2.1 Study population and clinical data collection

Between 2017 and 2021, a total of 1,262 high-risk breast cancer patients with suspected hereditary breast cancer syndromes were consecutively recruited from Zhejiang Cancer Hospital, Zhejiang Province, eastern China, based on comprehensive clinical criteria indicative of hereditary breast cancer syndromes. Eligibility was determined by the presence of any of the following: (1) early onset breast cancer diagnosed at ≤45 years of age; (2) multiple primary tumors (≥2) diagnosed at ≤50 years of age; (3) patients with one or more of the following family history characteristics: diagnosed at ≤50 years of age with a family history of one or more first-degree relatives with breast cancer, patients diagnosed at any age with two or more first-degree relatives diagnosed with breast cancer diagnosed at ≤50 years of age, at least two first-degree relatives with either pancreatic or prostate cancer (Gleason score ≥7), or the presence of male breast cancer in a first-degree relative; (4) patients diagnosed with triple-negative breast cancer (TNBC) at ≤60 years of age; and (5) male breast cancer diagnosed in the patient. Family pedigrees were constructed over three generations to validate the familial associations. Clinical and pathological data, including tumor size, lymph node status, estrogen receptor (ER) status, progesterone receptor (PR) status, human epidermal growth factor receptor 2 (HER2) expression, histologic grading, and menopausal status, were retrieved from the medical records and verified by certified pathologists. ER, PR, and HER2 status were evaluated using immunohistochemistry (IHC), as suggested by the American Society of Clinical Oncology/College of American Pathologists (ASCO/CAP). All data collected were anonymized and processed using Research Electronic Data Capture (REDCap), a secure and standardized electronic platform for data collection and management in clinical research.

### 2.2 Genomic DNA extraction and quality control

Genomic DNA was extracted from EDTA-anticoagulated peripheral venous blood samples using the QIAamp DNA Blood Mini Kit (Qiagen, Germany). DNA concentration, purity, and integrity were assessed by Qubit fluorometry, OD260/280 measurement, and agarose gel electrophoresis. In total, 1,262 samples with an OD260/280 ratio between 1.8 and 2.0 and a total DNA yield of more than 500 ng (Qiagen, 2024) were included in the downstream analyses.

### 2.3 Library preparation and target enrichment using a 98-gene panel

The Covaris M220 ultrasonicator was used to shear genomic DNA into fragments of 180–250 base pairs. The NEBNext Ultra II DNA Library Prep Kit (New England Biolabs, Ipswich, MA, USA) was used to perform end repair, A-tailing, and adapter ligation with Unique Molecular Identifiers (UMIs). Target enrichment was performed using a custom-designed 98-gene hereditary cancer panel based on the NEB target enrichment system, with probes synthesized by New England Biolabs (Ipswich, MA, USA). This panel covered complete exons and ±10 bp intronic flanking sequences (details of the genes are shown in **Supplementary Table 1**). This panel covered 27 breast cancer susceptibility genes (e.g., *BRCA1*, *BRCA2*, *PALB2*, and *ATM)*, and hybrid capture was applied to enhance specificity and sequencing depth. Following amplification and purification with AMPure XP beads, libraries were assessed for size distribution using an Agilent Bioanalyzer, and for purity and concentration using Qubit and quantitative PCR (qPCR).

### 2.4 High-throughput sequencing on the Illumina platform

Pooling of quantified libraries was performed on an equimolar basis, and sequencing was performed using paired-end (2 × 150 bp) runs on the Illumina HiSeq X Ten platform. Sequencing was designed to achieve an average target depth of 500× per sample. The quality metrics are shown in **Supplementary Table 2**. The read length and coverage uniformity were optimized to ensure confident and comprehensive variant calling across all target regions.

### 2.5 Bioinformatics pipeline and data preprocessing

Raw reads in FASTQ format were quality-checked using FastQC and summarized using MultiQC. Adapter sequences and low-quality bases were trimmed using Trim Galore. Clean reads were aligned to the human reference genome (GRCh38) using BWA-MEM v0.7.17. Post-alignment processing, including duplicate removal and UMI-based grouping, was conducted using Fgbio (Fulcrum Genomics). GATK v4.2 was used for BAM processing, including sorting, indexing, and base quality score recalibration.

### 2.6 Variant calling, filtering, and annotation

**Figure 1:**
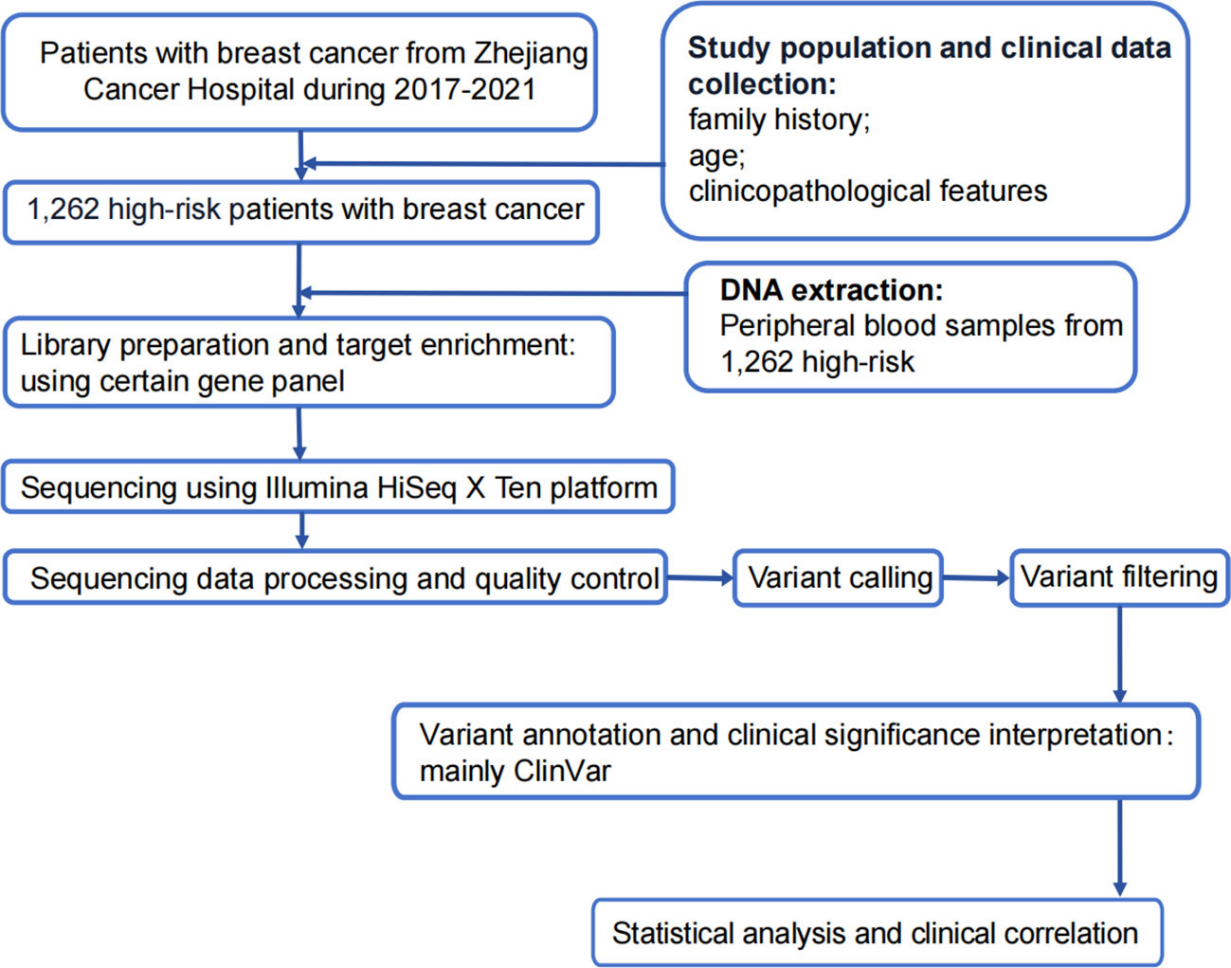
Workflow of genetic analysis and variant interpretation in high-risk breast cancer patients.

Variants were called using GATK HaplotypeCaller and FreeBayes, followed by variant filtering. The thresholds were set at QD > 2.0 and FS < 200 (GATK) and QUAL > 10 (FreeBayes). The VCF files from both callers were merged using bcftools. Annotations were performed using ANNOVAR and the Ensembl Variant Effect Predictor (VEP). Variant clinical significance was interpreted primarily using ClinVar clinical significance annotations (CLNSIG; accessed 25 June 2025) within the ACMG–AMP 2015 framework. Population frequency data from gnomAD EAS and in silico prediction results from SIFT, PolyPhen-2, MutationTaster, and CADD were used as supporting evidence. Variants in *BRCA1* and *BRCA2* were annotated and reported using Human Genome Variation Society (HGVS) nomenclature based on the MANE Select transcripts NM_007294.4 and NM_000059.4, respectively, following HGVS recommendations. **Figure 1** summarizes the overall workflow.

### 2.7 Mapping of variants observed in multiple participants

After annotation was performed using ClinVar as the primary reference, supplemented with in silico prediction tools for additional evidence, we summarized *BRCA1/2* P/LP variants observed in more than one participant. Given prior reports indicating inconsistencies in ClinVar annotations, particularly for underrepresented populations, we further compared our results with allele frequency data from gnomAD EAS. Since genomic data from Chinese populations are not publicly accessible, gnomAD EAS was used as the closest ethically appropriate available reference in our study (7).

### 2.8 Statistical analysis and clinical correlation

Associations between genetic variants and clinicopathological features were examined using appropriate statistical tests, including Fisher’s exact test and the chi-square test. A significance level of *p* < 0.001 was applied. All statistical analyses and visualizations were performed using R (version 4.5.0).

## 3 Results

### 3.1 Clinicopathological characteristics of the study population

We identified 81 *BRCA1/2* pathogenic or likely pathogenic (P/LP) variants in 124 (9.8%) cases, with 70 (5.5%) and 54 (4.3%) cases carrying *BRCA1* and *BRCA2* (42 vs. 39 variants), respectively. As shown in **Table 1**, 336 patients with TNBC were included, with 60 cases carrying *BRCA1/2* P/LP variants (51 and 9 cases carrying *BRCA1* and *BRCA2* variants, respectively). Although *BRCA1/2-*positive patients were slightly younger at diagnosis than *BRCA1/2-*negative patients (40.7 ± 11.2 vs 41.9 ± 10.0 years), this difference was not substantial. The molecular subtype showed significant differences, with a different distribution between groups (p < 0.001). In addition, as shown in **Table 2**, among patients aged ≤45 years, the distribution of molecular subtype differed significantly between those with and without *BRCA1/2* P/LP variants (more details are provided in **Supplementary Figure 1**). **Supplementary Table 3** shows that molecular subtype distribution differed between *BRCA1* and *BRCA2* P/LP variant carriers, with TNBC enriched among *BRCA1* carriers.

**Table 1:** Clinicopathological characteristics of 1,262 patients stratified by *BRCA1/2* variant status.

Characteristics	n	*BRCA1/2*+ (n = 124)	*BRCA1/2*- (n = 1,138)	*P*
Age of onset				
Mean		40.7 ± 11.2	41.9 ± 10.0	
≤ 45	831	82 (66.1%)	749 (65.8%)	0.881
> 45	427	41 (33.1%)	386 (33.9%)	
Lymph node metastasis				0.300
Yes	616	53 (42.8%)	563 (49.5%)	
No	600	62 (50.0%)	538 (47.3%)	
Distant metastasis				0.843
Yes	77	8 (6.5%)	69 (6.1%)	
No	1,185	116 (93.5%)	1,069 (93.9%)	
Tumor grade				0.172
I	23	1 (0.8%)	22 (1.9%)	
II	398	33 (26.6%)	365 (32.1%)	
III	389	47 (37.9%)	342 (30.1%)	
Molecular subtype				*p* < 0.001
Luminal A	514	36 (29.0%)	478 (42.0%)	
Luminal B	164	5 (4.0%)	159 (14.0%)	
HER2 overexpressing	126	3 (2.4%)	123 (10.8%)	
TNBC	336	60 (48.4%)	276 (24.3%)	

Information on the unknown cases was not listed. *BRCA1/2*+, *BRCA1/2* P/LP variants; *BRCA1/2-*, no *BRCA1/2* P/LP variants. P values compare *BRCA1/2*+ vs *BRCA1/2*-. Age was presented as mean ± SD. Variables were compared using the chi-square test or Fisher’s exact test.

**Table 2:** Clinicopathological characteristics of 1,262 patients stratified by age.

	≤ 45			> 45		
Characteristics	*BRCA1/2*+ (n = 82)	*BRCA1/2*- (n = 749)	*P*	*BRCA1/2*+ (n = 41)	*BRCA1/2*- (n = 386)	*P*
Lymph node metastasis			0.450			0.383
Yes	35 (42.7%)	369 (49.3%)		17 (41.5%)	193 (50.0%)	
No	40 (48.8%)	351 (46.9%)		22 (53.7%)	186 (48.2%)	
Distant metastasis			0.644			1.000
Yes	6 (7.3%)	48 (6.4%)		2 (4.9%)	21 (5.4%)	
No	76 (92.7%)	701 (93.6%)		39 (95.1%)	336 (87.0%)	
Tumor grade			0.186			0.733
I	1 (1.2%)	17 (93.6%)		0 (0%)	5 (1.3%)	
II	20 (24.4%)	238 (31.8%)		12 (29.3%)	127 (32.9%)	
III	31 (37.8%)	217 (29.0%)		16 (39.0%)	125 (32.4%)	
Molecular subtype			*p* < 0.001			0.200
Luminal A	25 (30.5%)	338 (45.1%)		11 (26.8%)	140 (36.3%)	
Luminal B	3 (3.7%)	129 (17.2%)		2 (4.9%)	30 (7.8%)	
HER2 overexpressing	2 (2.4%)	81 (10.8%)		1 (2.4%)	42 (10.9%)	
TNBC	41 (50.0%)	143 (19.1%)		18 (43.9%)	131 (33.9%)	

Information on the unknown cases was not listed. *BRCA1/2*+, *BRCA1/2* P/LP variants; *BRCA1/2−*,no *BRCA1/2* P/LP variants. P values compare *BRCA1/2*+ vs *BRCA1/2*−. Age was presented as mean ± SD. Variables were compared using the Chi-square test or Fisher’s exact test.

### 3.2 Computational and clinical annotation of *BRCA1/2* variants

The clinical significance of *BRCA1/2* variants was primarily interpreted based on ClinVar clinical significance annotations (CLNSIG; accessed 25 June 2025), within the framework of the ACMG-AMP 2015 guidelines. In silico prediction results were used as supporting evidence for variant interpretation. The majority of *BRCA1/2* variants in our cohort were classified as benign/likely benign (B/LB) (87.5%), demonstrating broad coverage of non-pathogenic variant classification in existing databases. Intermediate categories, such as conflicting interpretations of pathogenicity (0.5%), indicated inconsistencies in current reporting practices and complicated downstream interpretations. Clinically relevant subsets included P/LP variants (0.9%), which required further intervention clinically. The computational predictors (SIFT, PolyPhen-2, and MutationTaster) were also used as supporting evidence for the potential functional impact of variants; the results are provided in **Supplementary Table 4**.

### 3.3 Distribution and frequency of *BRCA1/2* variants

Based on the predicted results, across the entire cohort, synonymous variants accounted for 53.6% of all variants, followed by non-synonymous variants (44.5%). Other categories were much less frequent, including frameshift deletions (0.7%), intronic variants (0.5%), stop-gain variants (0.3%), and several rare types such as non-frameshift deletions, non-frameshift substitutions, non-frameshift insertions, and frameshift substitutions (<0.1% each). The most frequently observed synonymous variants were classified as B/LB (92.2%).

**Figure 2:**
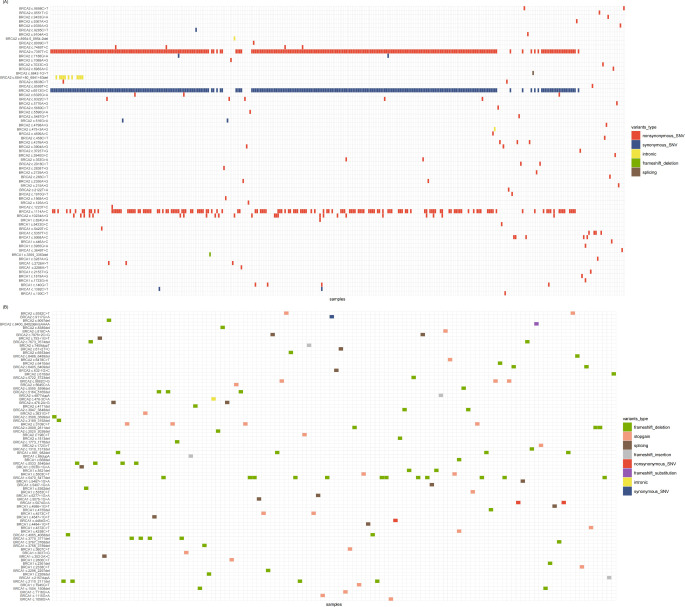
**(A)** Heatmap visualization of *BRCA1/2* variants in 329 samples with variants of uncertain significance (VUSs), identified through ANNOVAR and ClinVar annotation. **(B)** Heatmap visualization of variants in 124 samples with pathogenic or likely pathogenic (P/LP) variants, identified through ANNOVAR and ClinVar annotation.

A total of 13,265 variant-sample pairs were identified. The number of variants per patient in the cohort ranged from 1 to 11. In addition, **Figure 2a** showed variants of uncertain significance (VUSs) detected across 329 samples (29 cases of *BRCA1* and 310 of *BRCA2*). **Figure 2b** showed P/LP variants detected across 124 samples for P/LP, with 70 and 54 cases carrying *BRCA1* and *BRCA2* variants, respectively, as shown in **Supplementary Figure 2**, where frameshift deletions and stop-gain variants were the most frequent variant types, with *BRCA1* c.5470_5477del being the most frequent (12 patients).

In total, 21 P/LP variants observed in more than one patient were initially identified using ClinVar annotation. There were 11 P/LP variants observed in *BRCA1*, including c.5470_5477del (12 patients, 1.0% of the cohort), c.3770_3771del (4, 0.3%), c.981_982del (4, 0.3%), c.2110_2111del (4, 0.3%), c.5503C>T (2, 0.2%), c.4065_4068del (2, 0.2%), c.5521del (2, 0.2%), c.1504_1508del (2, 0.2%), c.4573C>T (2, 0.2%), and c.5074G>A (2, 0.2%), c.5533_5540del (4, 0.3%), c.1504_1508del (2, 0.2%). There were 10 P/LP variants observed in *BRCA2*, including c.3109C>T (4, 0.3%), c.5682C>G (3, 0.2%), c.2808_2811del (3, 0.2%), c.5164_5165del (3, 0.2%), c.476-3C>A (2, 0.2%), c.3847_3848del (3, 0.2%), c.9382C>T (2, 0.2%), c.6405_6409del (2, 0.2%), c.7673_7674del (2, 0.2%), and c.7976 + 2C>G (2, 0.2%).

### 3.4 Pathogenicity classification and distribution of *BRCA1/2* variants

**Figure 3:**
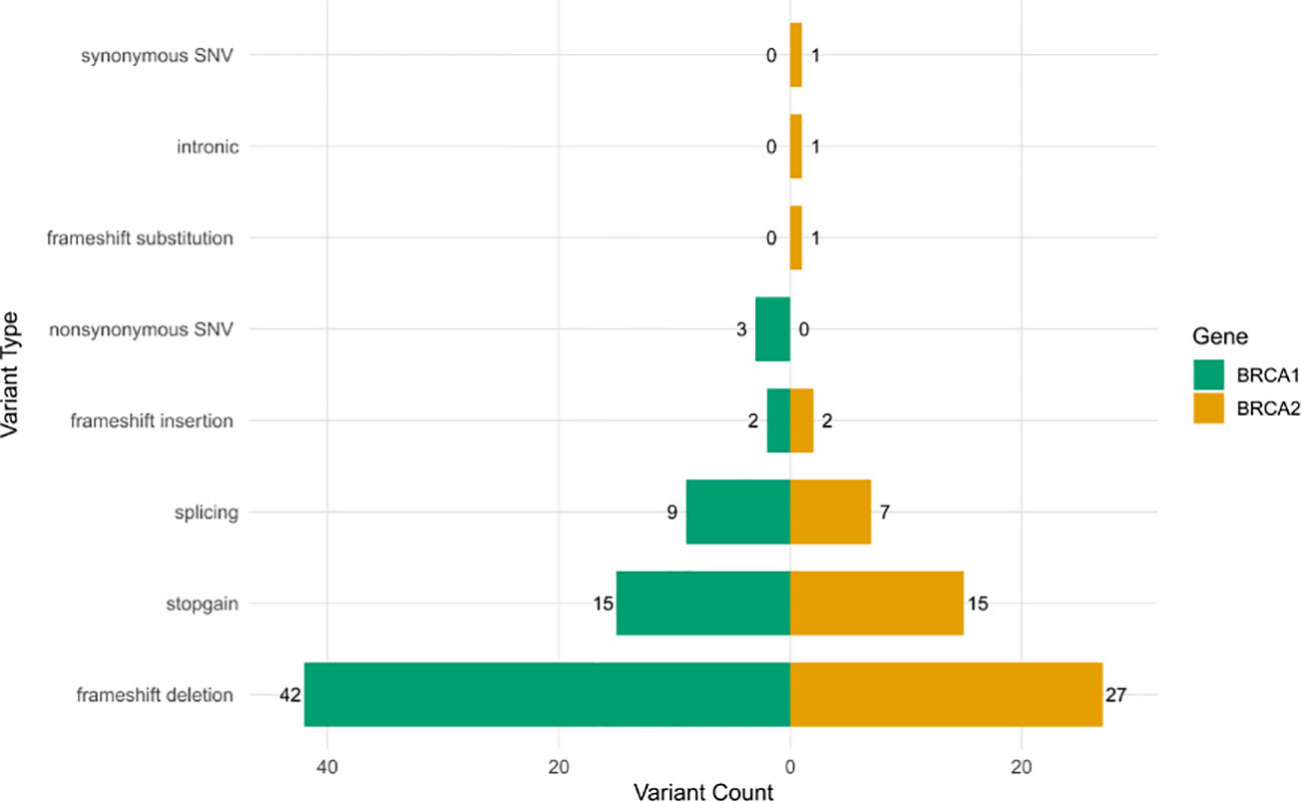
Distribution of *BRCA1/2* pathogenic or likely pathogenic (P/LP) variants identified through ANNOVAR and ClinVar annotation.

**Figure 4:**
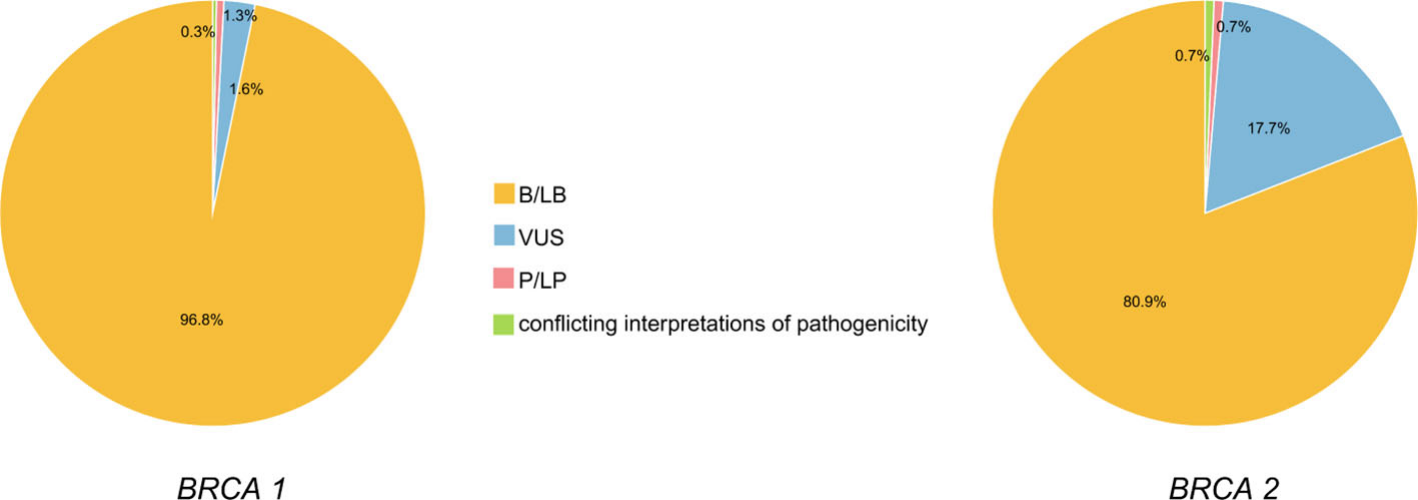
Distribution of ClinVar clinical significance categories among *BRCA1/2* variants.

**Figure 5:**
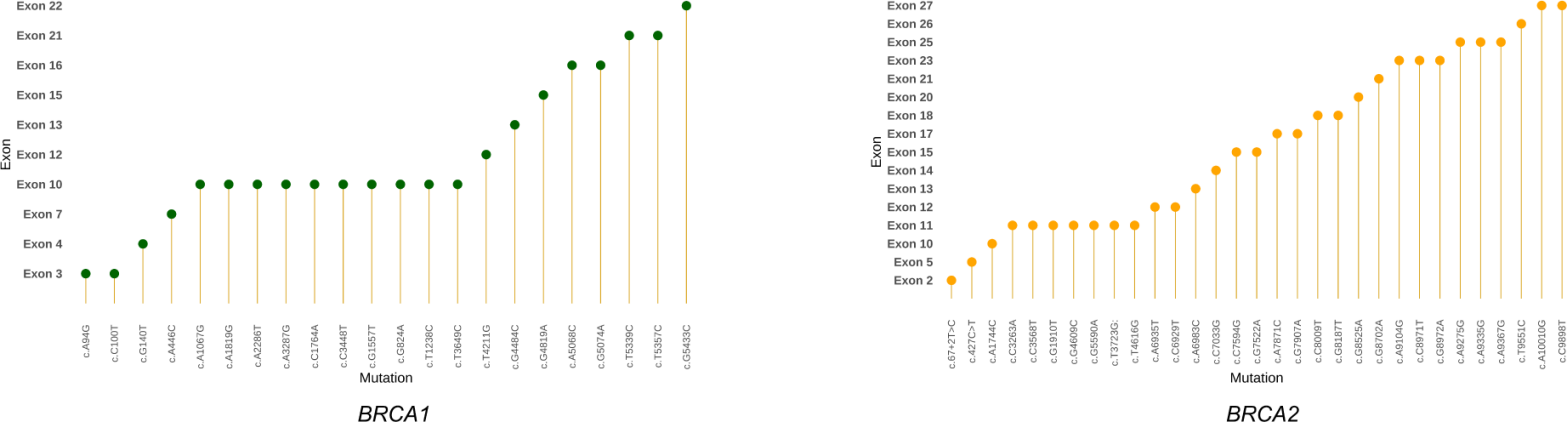
Lollipop plot of *BRCA1/2* pathogenic or likely pathogenic (P/LP) variants across exons.

*BRCA1/2* variants were annotated using ANNOVAR and the Ensembl VEP. Overall, the analyzed patient cohort contained 81 distinct P/LP variants in the *BRCA1/2* genes, comprising 42 variants in *BRCA1* and 39 variants in *BRCA2*. The distribution of variant types in our cohort was shown in **Figure 3**, where frameshift deletions variants constituted the most common functional class in both *BRCA1* and *BRCA2*, accounting for 49 and 87 variants, respectively. Frameshift deletions were the second most frequent category, with 26 in *BRCA1* and 36 in *BRCA2*. In addition, one *BRCA2 P/LP* variant, *BRCA2* c.9117G>A, was annotated as a synonymous variant. Although this variant did not alter the encoded amino acid, it affected the last nucleotide of exon 23 and had been reported to disrupt normal splicing, resulting in exon 23 skipping, premature termination, and a predicted non-functional *BRCA2* protein. Therefore, this variant was retained in the P/LP category based on ClinVar/ACMG-AMP interpretation rather than on protein-coding consequence alone. Our results for the ClinVar classifications were shown in **Figure 4**. A total of 0.3% and 0.7% of variants carried conflicting interpretations in *BRCA1* and *BRCA2*, respectively, whereas 1.6% and 17.7% of variants carried VUSs in *BRCA1* and *BRCA2*, respectively, underscoring the challenges and importance of reaching consensus in clinical interpretation. As shown in **Figure 5**, these variants were unevenly distributed across exons, with *BRCA1* variants mostly mapped to exon 10 and *BRCA2* variants mostly mapped to exon 11.

## 4 Discussion

This study analyzed germline *BRCA1/2* variants in 1,262 high-risk breast cancer patients from Zhejiang Cancer Hospital, eastern China, constituting the largest cohort of high-risk patients with breast cancer from mainland China reported to date. Overall, 124 patients carried 81 distinct *BRCA1/2* P/LP variants, including 70 *BRCA1* and 54 *BRCA2* P/LP variant carriers. Most *BRCA1/2* P/LP carriers were diagnosed at ≤45 years, and P/LP variants were enriched among patients with TNBC, with 60 of 336 TNBC patients carrying *BRCA1/2* P/LP variants. In addition, 21 recurrent P/LP variants were observed in more than one participant, among which *BRCA1* c.5470_5477del, a known founder mutation in Chinese populations, was the most frequent.

The overall *BRCA1/2* P/LP carrier rate in our cohort was 9.8%, which was lower than that reported in several previous Chinese high-risk cohorts, including 17.0% in a cohort of 937 patients (8), 18.3% in a cohort of 71 high-risk patients (9), and 17.4% in a multicenter cohort of 437 high-risk patients from 14 cities in China (10). These differences may be partly explained by heterogeneity in inclusion criteria, sample size, age distribution, pathological restrictions, and variant interpretation strategies. First, some previous cohorts included a higher proportion of very early-onset patients, such as those diagnosed at ≤35 years of age, with a lower average age than our cohort. Because early-onset breast cancer is associated with a higher probability of *BRCA1/2* P/LP variant carriage (11), differences in age composition may influence the reported carrier rate. Second, our study adopted a conservative variant interpretation strategy, using ClinVar clinical significance annotations as the primary reference within the ACMG–AMP framework. In addition, variants were identified as P/LP variants in the above studies according to two broader criteria: (1) the inclusion of certain variant types and (2) classification as pathogenic based on evidence from the published literature. These classification criteria may have resulted in broader inclusion of variants than the ClinVar-based approach used in our study. Therefore, the lower carrier rate observed in our cohort could be interpreted in the context of cohort design and variant classification strategy, rather than as a direct biological discrepancy. Among TNBC patients, the *BRCA1/2* P/LP carrier rate in our cohort was 17.9%, which was lower than the 23.4% reported in a high-risk cohort from northern China (10). This difference may also reflect differences in inclusion criteria and age distribution. Nevertheless, the enrichment of *BRCA1/2* P/LP variants among TNBC patients and patients diagnosed at ≤45 years of age was consistent with previous evidence from Chinese and Hong Kong high-risk cohorts (12). These findings support the clinical value of prioritizing genetic counseling and *BRCA1/2* testing in younger patients and patients with TNBC, especially in clinically selected high-risk populations (13).

We summarized 21 recurrent *BRCA1/2* P/LP variants that were observed in more than one participant. Available evidence suggests that several of these variants had been repeatedly reported in Chinese or Asian populations. *BRCA1* c.5470_5477del was the most frequent recurrent variant in our cohort, occurring in 12 patients, and had been reported as a founder mutation in Chinese populations (14). Its enrichment in Chinese and East Asian populations further supports its population-specific relevance (14, 15). Other *BRCA1* variants, including c.5521del, c.4573C>T, c.981_982del, and c.2110_2111del, had also been described in Chinese or broader Asian cohorts (16–18). Similarly, *BRCA2* c.3109C>T had been reported in Chinese and Pakistani patients and had been proposed as a potential founder mutation across Asian populations (18–20).

In addition to variants enriched in Chinese or Asian populations, several recurrent variants in our cohort had been reported across wider geographic regions. *BRCA1* c.1504_1508del had been documented in Türkiye, India, China, and populations from the Middle East, North Africa, and Southern Europe (21–23). *BRCA2* c.5682C>G, c.2808_2811del, c.3847_3848del, c.5164_5165del, c.476-3C>A, c.6405_6409del, and c.9382C>T had also been reported in Chinese, European, Middle Eastern, or other non-Asian populations (14, 24–32). These findings suggested that recurrent *BRCA1/2* P/LP variants observed in Chinese patients may reflect both population-specific enrichment and broader cross-population sharing. However, the founder status of several recurrent variants remained unsettled. *BRCA1* c.3770_3771del had been reported in Chinese, Malaysian, Spanish, and Pakistani cohorts, but whether it should be considered a shared founder mutation across Asian populations remains debated (16, 17, 33, 34). *BRCA1* c.4065_4068del had been recognized as a founder variant in Pakistan but had also been detected in Chinese Hakka and East African populations (19, 35–37). Therefore, determining whether these variants represent shared founder mutations across populations or independent recurrent events will require larger ancestry-matched datasets and family-based haplotype analyses (38).

The predominance of P/LP variants identified in *BRCA1* exon 10 and *BRCA2* exon 11 was likely attributable to the substantial length of these exons (39). This may partly reflect a length-driven effect rather than true mutational hotspots.

Our study provides a characterization of the *BRCA1/2* P/LP variant landscape in a clinically selected high-risk breast cancer cohort from Zhejiang Province, eastern China. By reflecting a real-world referral setting, our findings may help inform genetic counseling and testing prioritization for clinically high-risk patients. Importantly, timely identification of germline *BRCA1/2* status is not only relevant for risk assessment but may also affect subsequent clinical management. Germline *BRCA* testing could provide actionable information for surgical planning, including decisions regarding contralateral risk-reducing procedures and the potential reduction of staged surgeries in appropriate patients (40, 41). Germline *BRCA* status also has direct therapeutic implications, particularly with respect to PARP inhibitor–based treatment strategies in eligible patients (42, 43). In China, where genetic testing is not consistently covered by public insurance and is often self-paid, these data may provide a practical reference for prioritizing testing in clinically high-risk patients.

Our study has several strengths. First, although large unselected cohorts in China have provided information on *BRCA1/2* P/LP variants at the population level, our clinically selected high-risk cohort offers complementary characterization that was more closely aligned with current clinical testing pathways. In addition, this study represents the largest high-risk cohort reported to date in mainland China, highlighting the need for Chinese-specific resources characterizing the *BRCA1/2* variant landscape. This study also has some limitations. As the cohort was derived from a single cancer center without long-term follow-up, it may not fully capture the diversity of the broader Chinese population. In addition, because family-based haplotype information was unavailable, validation of the effects of the multiple-observed P/LP variants will require studies in larger cohorts with integrated pedigree data.

In summary, we analyzed *BRCA1/2* variants in a large high-risk Chinese breast cancer cohort and provide a comprehensive characterization aligned with current clinical testing pathways. By generating population-specific reference data for high-risk patients, our findings may serve as a valuable reference for optimizing genetic screening strategies and risk management in clinical practice in eastern China. Nevertheless, further validation in larger, independent multicenter cohorts, together with functional studies, will be essential to confirm the pathogenicity of variants and to strengthen the clinical utility of these findings.

## Data Availability

The sequencing data generated in this study are not publicly available due to legal, ethical, and privacy restrictions related to human genetic resources and patient confidentiality. The original contributions presented in the study are included in the article/Supplementary Material. Requests to access the datasets should be directed to the corresponding author and will be considered subject to institutional ethics approval and applicable regulatory requirements.
