# Supplementary material for: BRCA1/2 variant landscape and clinical correlates in high-risk breast cancer patients from Eastern China
Source: Front Oncol. 2026 Jun 29;16:1792634. doi: 10.3389/fonc.2026.1792634 (PMC13357219; doi:10.3389/fonc.2026.1792634)
Supplement: Supplementary file 1 [file DataSheet1.zip › Supplementary_June11/Supplementary Figures.pdf]

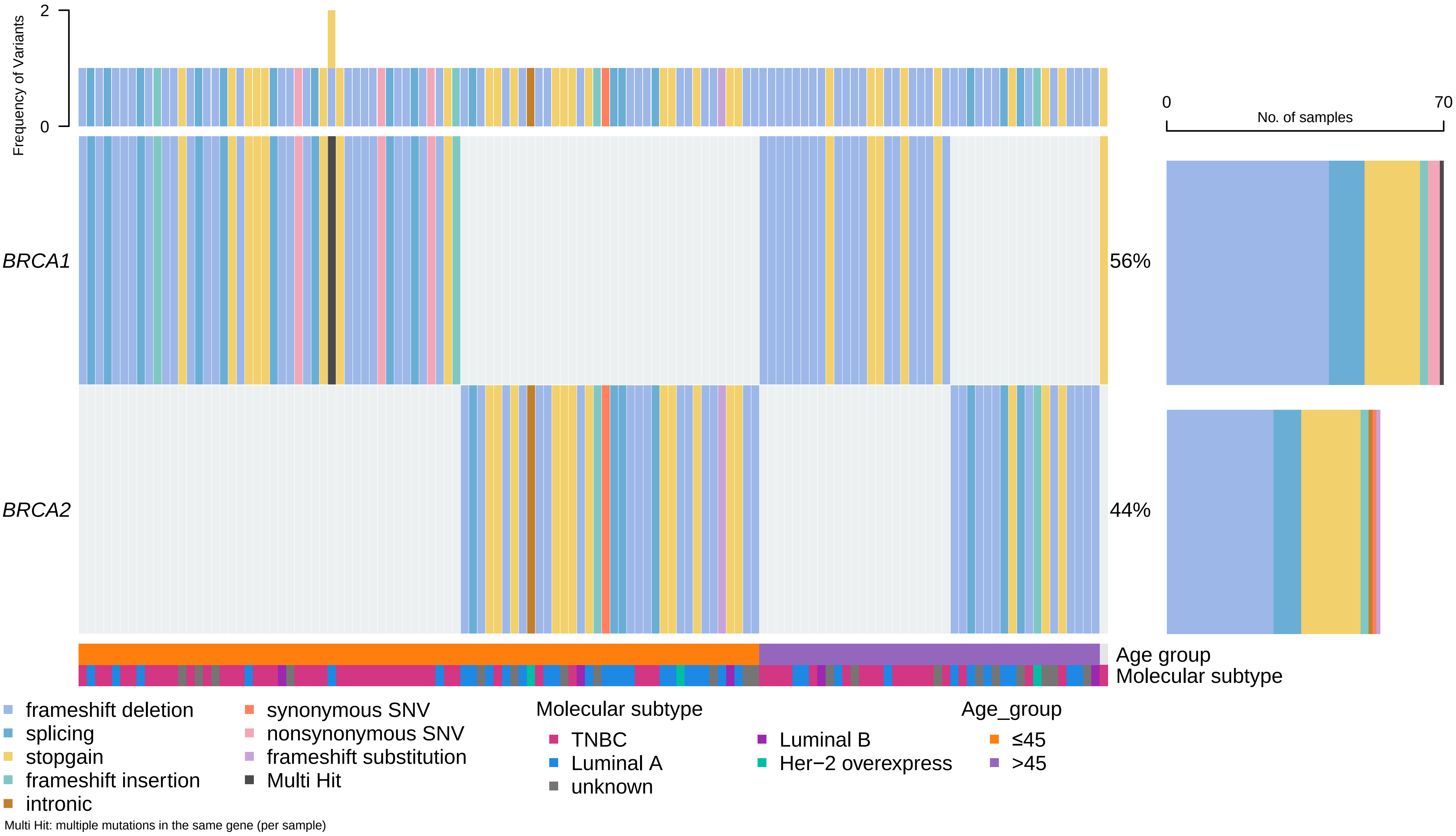

Supplementary Figure 1. Distribution of *BRCA1/2* pathogenic or likely pathogenic (P/LP) variant carriers among high-risk breast cancer patients, stratified by age group and molecular subtype.

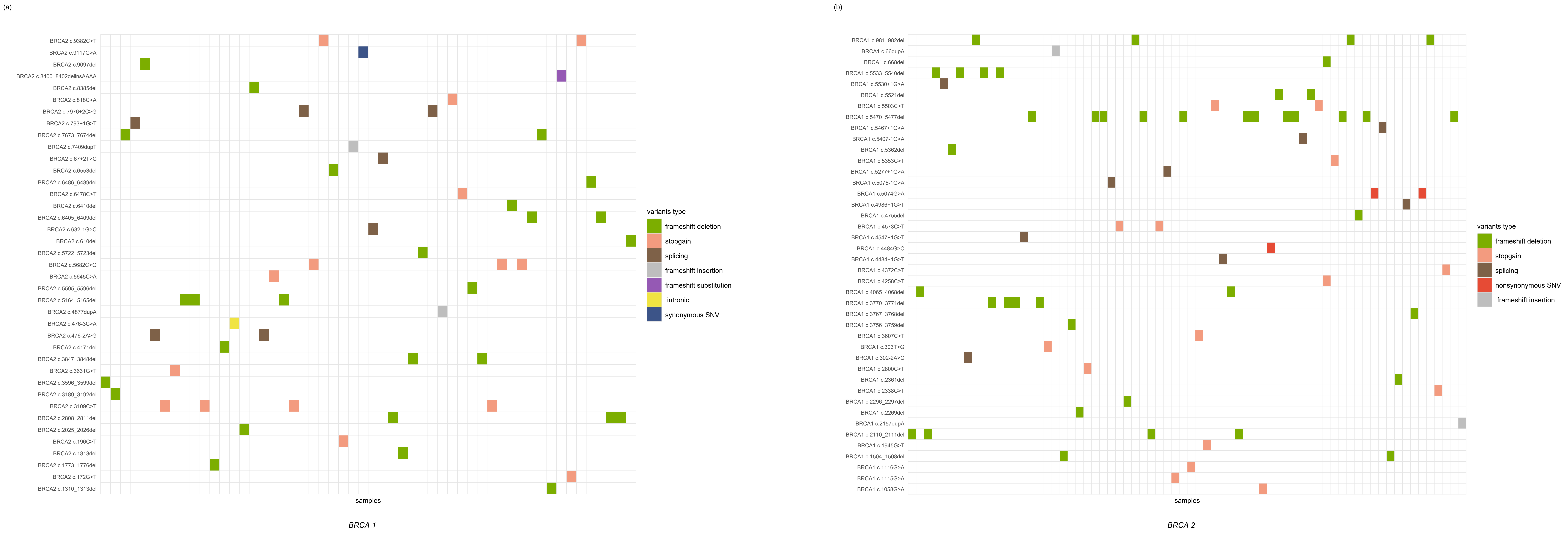

Supplementary Figure 2. (A) Distribution of *BRCA1* P/LP variants in 70 Breast Cancer Samples. (B) Distribution of *BRCA2* P/LP variants in 54 Breast Cancer Sample.
