# Supplementary material for: BRCA1/2 variant landscape and clinical correlates in high-risk breast cancer patients from Eastern China
Source: Front Oncol. 2026 Jun 29;16:1792634. doi: 10.3389/fonc.2026.1792634 (PMC13357219; doi:10.3389/fonc.2026.1792634)
Supplement: Supplementary file 1 [file DataSheet1.zip › Supplementary_June11/Supplementary.DOCX]

Supplementary Material

Supplementary Figure 1. Distribution of *BRCA1/2* pathogenic or likely pathogenic (P/LP) variant carriers among high-risk breast cancer patients, stratified by age group and molecular subtype.

Supplementary Figure 2. (A) Distribution of *BRCA1* P/LP variants in 70 Breast Cancer Samples. (B) Distribution of *BRCA2* P/LP variants in 54 Breast Cancer Sample.

Supplementary Table 1. Lists of 98 genes..

Supplementary Table 2. The quality metrics of sequencing.

Supplementary Table 3. Clinicopathological characteristics of 124 patients stratified by *BRCA1/2* mutation status.

Supplementary Table 4. Clinical significance and in silico predictions of *BRCA1/2* variants.
